# Correction: Intermittent Hypoxia Effect on Osteoclastogenesis Stimulated by Neuroblastoma Cells

**DOI:** 10.1371/journal.pone.0170156

**Published:** 2017-01-13

**Authors:** Vasantha Kumar Bhaskara, Indra Mohanam, Meena Gujrati, Sanjeeva Mohanam

Fig 4 has numerous errors. Fig 4B column 2 row 2 (image: U0126, 1.0 μM) was inverted (top to bottom). Fig 4B column 2 row 3 (image: SP600125, 5.0 μM) was duplicated in column 1 row 4 (image under SB203580, 0.1 μM). Please see the correct Fig 4 here.

**Fig 4:**
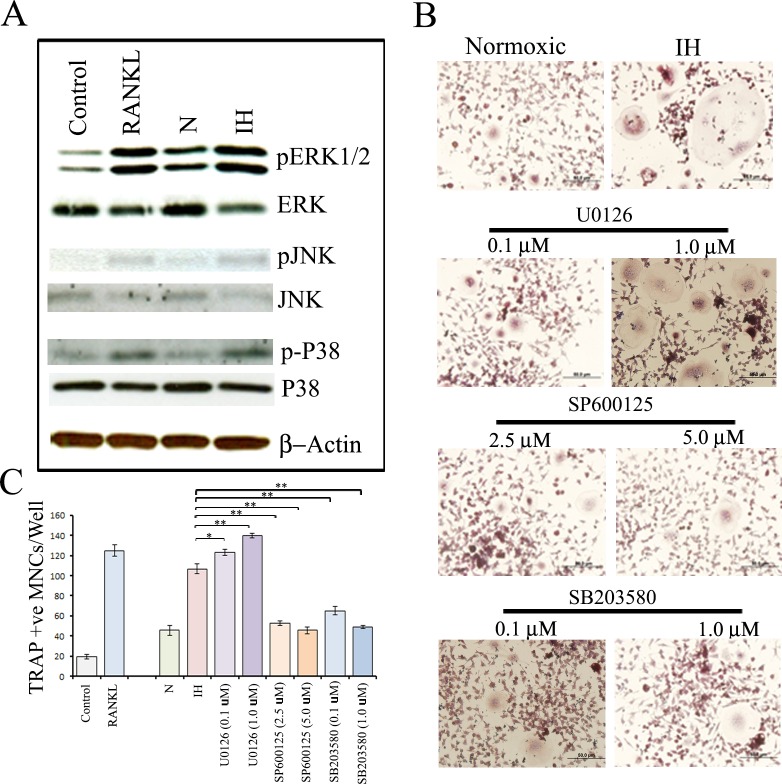
Effects of intermittent hypoxia exposure to neuroblastoma cells on modulation of osteoclastogenic signaling pathways in RAW 264.7 cells. (A) RAW 264.7 cells were exposed to growth medium (control), growth medium with RANKL (100 ηg/ml), CM from parental SH-SY5Y cells, or intermittent hypoxia-exposed (IH) cells. The cells were then allowed to grow for six days under normoxia with medium change after three days. The JNK, ERK and p38 activation states were determined by immunoblot analysis using antibodies specifically directed against the phosphorylated forms of the enzymes, compared to data obtained with antibodies directed against the unphosphorylated states of the kinases. (B) RAW 264.7 cells were exposed to CM from parental SH-SY5Y cells (N), IH cells or IH cells treated with 0.1 or 1 μM U0126 (MEK signaling pathway inhibitor), 2.5 or 5 μM SP600125 (JNK signaling pathway inhibitor) and 0.1 or 1 μM SB203580 (p38 signaling pathway) and cultured under normoxia. The medium was changed after three days. After six days, cultures were analyzed for TRAP-positive multinucleated cells containing three or more nuclei under a light microscope. A representative image is shown. (C) Graphical representation of the number of TRAP-positive multinucleated cells. Values represent the number of TRAP positive cells counted in ten different fields for each experiment from three independent experiments and expressed as mean ± SD. *P<0.05, **P<0.01 IH versus IH treated with SP600125 (2.5 or 5 μM) or SB203580 (0.1 or 1 μM).
